# Next-Generation Sequencing on Insectivorous Bat Guano: An Accurate Tool to Identify Arthropod Viruses of Potential Agricultural Concern

**DOI:** 10.3390/v11121102

**Published:** 2019-11-28

**Authors:** Mathieu Bourgarel, Valérie Noël, Davies Pfukenyi, Johan Michaux, Adrien André, Pierre Becquart, Frédérique Cerqueira, Célia Barrachina, Vanina Boué, Loïc Talignani, Gift Matope, Dorothée Missé, Serge Morand, Florian Liégeois

**Affiliations:** 1 Animal Santé Territoire Risque Environnement- Unité Mixe de Recherche 117 (ASTRE) Univ. Montpellier, Centre International de Recherche Agronomique pour le Développement (CIRAD), Institut National de la Recherche Agronomique, 34398 Montpellier, France; mathieu.bourgarel@cirad.fr (M.B.); serge.morand@cirad.fr (S.M.); 2 Centre International de Recherche Agronomique pour le Développement (CIRAD), Research Platform-Production and Conservation in Partership, Unité Mixe de Recherche ASTRE, Harare, Zimbabwe; 3 Maladies Infectieuses et Vecteurs: Ecologie, Génétique, Evolution et Contrôle- Unité Mixe de Recherche 224 (MIVEGEC), Institut de Recherche pour le Développement (IRD), Centre National de Recherche Scientifique (CNRS), Univ. Montpellier, 34398 Montpellier, France; valerie.noel@ird.fr (V.N.); pierre.becquart@ird.Fr (P.B.); svetnina@yahoo.fr (V.B.); loic.talignani@ird.fr (L.T.); dorothee.misse@ird.fr (D.M.); 4 Faculty of Veterinary Science, University of Zimbabwe, P.O. Box MP167, Mt. Pleasant Harare P.O. Box MP167, Zimbabwe; dpfukas@gmail.com (D.P.); gmatope@vet.uz.ac.zw (G.M.); 5 Université de Liège, Laboratoire de Génétique de la Conservation, GeCoLAB, 4000 Liège, Belgium; johan.michaux@ulg.ac.be (J.M.); adrien.andre@uliege.be (A.A.); 6 Institut des Sciences de l’Evolution de Montpellier (ISEM), Univ Montpellier, Centre National de Recherche Scientifique (CNRS), Ecole Pratique des Hautes Etude (EPHE)s, Institut de Recherche pour le Développement (IRD), 34398 Montpellier, France; frederique.cerqueira@umontpellier.fr; 7 Montpellier GenomiX (MGX), Biocampus Montpellier, Centre National de Recherche Scientifique (CNRS), Intitut National de la Santé et de la Recherche Médicale (INSERM), Univ Montpellier, 34094 Montpellier, France; celia.barrachina@mgx.cnrs.fr; 8 Institut des Sciences de l’Evolution de Montpellier (ISEM) Univ. Montpellier, Centre National de Recherche Scientifique (CNRS), Institut de Recherche pour le Développement (IRD), Centre International de Recherche Agronomique pour le Développement (CIRAD), 34000 Montpellier, France

**Keywords:** Dicistrovirus, Bats, Faeces, Phylogeny, HTS, Zimbabwe

## Abstract

Viruses belonging to the *Dicistroviridae* family have attracted a great deal of attention from scientists owing to their negative impact on agricultural economics, as well as their recent identification as potential aetiological agents of febrile illness in human patients. On the other hand, some Dicistroviruses are also studied for their potential biopesticide properties. To date, Dicistrovirus characterized in African mainland remain scarce. By using High-Throughput Sequencing technology on insectivorous bat faeces (*Hipposideros Caffer*) sampled in a cave used by humans to collect bat guano (bat manure) as fertilizer in Zimbabwe, we characterized the full-length sequences of three *Dicistrovirus* belonging to the *Cripavirus* and *Aparavirus* genus: *Big Sioux River Virus-Like* (*BSRV*-Like), *Acute Bee Paralysis Virus* (*ABPV*), and *Aphid Lethal Paralysis Virus* (*ALPV*). Phylogenetic analyses of ORF-1 and ORF-2 genes showed a complex evolutionary history between *BSRV* and close viruses, as well as for the *Aparavirus* genus. Herewith, we provide the first evidence of the presence of *Dicistrovirus* in Zimbabwe and highlight the need to further document the impact of such viruses on crops, as well as in beekeeping activities in Zimbabwe which represent a crucial source of income for Zimbabwean people.

## 1. Introduction

*Dicistroviridae* is a family of non-enveloped viruses with a linear ssRNA genome of approximately 7–10 kb. The *Dicistrovirus* RNA genome contains two non-overlapping open reading frames (ORFs) separated by an Inter-Genic Region (IGR) internal ribosome entry site (IRES) [1]. In the absence of 5′cap, Dicistroviral RNA is translated by means of an IRES. The ORF-1 encodes for the non-structural proteins, whereas the ORF-2 encodes for structural proteins. Replication occurs in the cytoplasm of an infected cell. According to the International Committee on Taxonomy of Viruses (ICTV), the *Discistrovirus* comprises three different genera: the *Triatovirus*, *Aparavirus,* and *Cripavirus* [2].

All classified *Dicistrovirus* members infect arthropod hosts, and some of them can wreak havoc in beehives (e.g., the *Acute bee paralysis virus* and *Israeli acute paralysis* virus, which infect domesticated bees) or in shrimp and crab farming (e.g., the *Taura syndrome virus*, *Macrobrachium rosenbergii virus*, *Mud crab dicistrovirus*) and might have a devastating economic impact [3,4,5,6,7,8]. Besides, some dicistroviruses are pathogenic to insect pests of agricultural or medical importance, rendering them as potentially interesting biopesticides [3]. *Dicistroviruses* are likely to be ubiquitous and have been identified in multiple environments [9,10].

The transmission of the *Dicistrovirus* can occur in different ways: horizontally, per os, via viral particles present in the faeces of infected arthropods from females to males [11] or via plant-mediated transmission [12], and vertically, by transovarian or transovum transmission [13,14,15]. Additionally, the Dicistrovirus can also be transmitted through vectors such as Varroa’s mite [16].

Beyond the classified *Dicistrovirus* species, numerous dicistroviruses which are yet to be classified have been characterized in the last two decades mainly owing to the advent of high-throughput sequencing technologies (HTS). Although numbers of them were identified in insects [12,17,18,19,20,21,22], some were found in mammalian stools, including human beings. However, the presence of such viruses in mammalian faeces were likely linked to their dietary habits [23,24,25,26,27,28].

Recently, blood-associated Dicistroviruses were described in both bats and humans [29,30,31]. Notably, in Tanzania, Dicistrovirus genome sequences were detected in 103 out of 670 (15.3%) sera tested from febrile Tanzanian children. More interestingly, these results were confirmed by real-time RT-PCR in 30 out of the 38 sera tested with a median of 5.7 x 10^3^ (1.32 X 10^3^- 1.44 X 10^7^) viral RNA copies/mL of sera [30]. Although this study did not allow to establish a clear link between this viral infection and the febrile state of children, it highlighted the need to further document the potential spillover of these viruses from their arthropod hosts to mammals, and particularly in human beings. However, in order to assess the human’s viral exposure risk, it is crucial to characterize circulating viruses in human working environments, especially where interface with wildlife is involved.

In this study, by using HTS technologies on a pool of insectivore bat faeces collected in a cave known to be regularly used by humans, we provide the first evidence of the *Big Sioux River Virus-like* (*BSRV-like*), *Aphid Lethal Paralysis Virus* (*ALPV*), and *Acute Bee Paralysis Virus* (*ABPV*) circulation in Zimbabwe.

## 2. Materials and Methods

### 2.1. Collection of Samples

Between June 2016 and February 2017, insectivorous bat (*Hipposideros caffer*) faecal samples were collected in the Magweto cave (S17.10024°, E029.19214°), which is regularly visited by local communities to collect bat guano, which they use as fertilizer [31]. Two square meters of plastic sheets were laid down in the cave underneath the bat colonies overnight (five plastic sheets per cave). Faeces were collected from each plastic sheet at a rate of ≈ 6 g of pooled faeces in a 15 mL tube containing 6 mL of home-made RNA stabilization solution which allowed for avoidance of RNA degradation during the field mission [32]. Samples were stored at –80 °C until laboratory analyses.

### 2.2. Species Identification

Bat species were identified by Cytochrome b amplification [33] and sequencing after DNA extraction using the Qiamp DNA stool (Qiagen S.A, Courtaboeuf, France). Cytochrome b sequences were then compared to the bat sequences available in the GenBank database using the Basic Local Alignment Search Tool (BLAST) program, and species were confirmed by phylogenetic analysis (Figure S1). Only bats from *Hipposideros* spp. were identified. Following the current literature, only two different *Hipposideros* bat species have been reported in Zimbabwe to date: *Hipposideros caffer* and *Hipposideros vittatus* [34]. All our samples were closer to *H. caffer* than any other *Hipposideros* species [32].

### 2.3. Diet Identification

DNA was extracted from each sample with the QIAamp DNA Stool Mini Kit (Qiagen, Hombrechtikon, Switzerland). PCR amplification was duplicated for each sample on a portion of the mitochondrial cytochrome oxydase I gene [35]. Negative DNA extraction and negative PCR controls were included in the procedure. Agencourt AMPure XP beads (BeckmanCoulter Life Sciences, Indianapolis, IN, USA) and then the Quant-iTTMPicoGreen^®^dsDNA Assay Kit (Thermo Scientific, Waltham, MA, USA) were used to purify PCR products and to quantify purified amplicons, respectively. After the quantification step, products were pooled at equimolarity and sent to the GIGA Genomics platform (University of Liège, Belgium) for sequencing on an ILLUMINA NextSeq benchtop sequencer. Raw sequences were analyzed as described previously [36].

### 2.4. RNA Extraction

RNA extraction was carried out from a pool of faecal samples collected on the same plastic sheet. Four sample tubes from the same plastic sheet were pooled and transferred to a 50 mL tube with 10 mL of PBS 1X, then was vigorously mixed and centrifuged at 4500 rpm for 10 min (Centrifuge Jouan GR4i). The supernatant was filtered using gauze in order to eliminate faecal matter, and transferred into fresh tubes before re-centrifuging at 4500 rpm for 10 min (Centrifuge Jouan GR4i). The supernatant was filtered through a 0.45 µm filter to remove eukaryotic and bacteria-sized particles. Seven milliliters of the filtered samples were centrifuged at 250,000 g for 2.5 h at 4 °C. The pellets were re-suspended in 600 µl molecular-grade H20, and 150 µl was used to extract RNA using the NucleoSpin^®^ RNA Kit (Macherey-Nagel, Hoerdt, France) according to the manufacturer’s protocol. Then, RNA was DNAse-treated by using the Turbo DNA-free kit (Thermo Fisher Scientific, Illlkirch France).

### 2.5. RNA Sequencing and rRNA Depletion

The RNA-Seq library was constructed with the Truseq stranded mRNA sample preparation (low-throughput protocol) kit from Illumina. One microgram of total RNA was used for the rRNA depletion using the Ribo-Zero rRNA Removal Kit (Illumina, San Diego, CA USA). Purified mRNAs were validated by capillary electrophoresis on a Fragment Analyzer (Advanced Analytical, Ankeny, IA, USA

### 2.6. Library Construction

Approximately 7.5 ng of purified mRNA were used for the library construction. They were fragmented into small pieces using divalent cations under elevated temperature. The cleaved RNA fragments were copied into first-strand cDNA using SuperScript II reverse transcriptase, Actinomycin D, and random hexamer primers. The second-strand cDNA was synthesized by replacing dTTP with dUTP. These cDNA fragments then had the addition of a single “A” base and subsequent ligation of the adapter. The products were then purified and enriched with 15 cycles of PCR. The final cDNA library was validated with a Fragment Analyzer (Advanced Analytical, Ankeny, IA, USA) and quantified with a KAPA qPCR kit (Kapa Biosystems, Wilmington, MA USA).

### 2.7. Library Sequencing

The library was denatured using NaOH and diluted to 12 pM with 5% of PhiX before loading on a MiSeq (Illumina, San Diego, CA USA). Cluster formation, primer hybridization, and sequencing of 300 cycles in the paired-end read were performed with a reagent kit v3 (600 cycles).

### 2.8. Bioinformatic Analyses

Illumina adapters were trimmed using MiSeq Reporter software. We obtained 8,680,414 paired reads. Quality was checked using FastQC software (bioinformatics.babraham.ac.uk/projects/fastqc/). Due to the low sequence quality per base in reads 2, only reads 1 were used. Reads were compared to the C-RVDBv12.2 viral database [37] using BLAST 2.6.0 [38] (command lines in Figure S2). Matching reads amounted to 524,042 (6.04% of total reads). There were 160,737 reads which were identified as Big Sioux River viruses, 796 as Acute Bee Paralysis viruses, and 282 as Aphid Lethal Paralysis viruses. A total of 161,815 Dicistrovirus reads (30.88% of viral reads) were identified. These reads were extracted (see command lines in Supplementary Materials Text S3) and analysed with Geneious software (Biomatters LTD, Auckland, NZ).

### 2.9. Polymerase Chain Reactions

The RNA extracted from the sample’s pool was reverse-transcribed using random hexamers. The ALPV, ABPV, and BSRV specific primers were designed and used to amplify the targeted viruses. PCR products were then agarose-gel purified (Geneclean Turbo Kit, MP Biomedicals, Illlkirch, France) and directly sequenced in both 5′ and 3′ directions using cycle sequencing and dye terminator methodologies (Eurofins, Ebersberg, Germany). Overlapping sequences were assembled into contiguous sequences using SEQMAN DNASTAR software (Lasergene, DNASTAR, Inc., Madison, WI, USA).

### 2.10. Genetic Analyses

Predicted ORF-1 and ORF-2 *Dicistrovirus* amino acid sequences were aligned using MEGA 7 [39], with minor manual adjustments. Sites that could not be unambiguously aligned were excluded, and divergent regions were excluded from subsequent analyses. Phylogenies were inferred using the Maximum Likelihood (ML) method implemented in PhyML [40]. The reliability of branching orders was tested using the bootstrap approach (1000 replicates). The suited evolution model (LG + Γ4 + I) was selected by Akaike’s Information criterion (AIC) using Topali software [41]. Identity analyses were done using ClustalX [42].

In order to study whether the newly characterized BSRV-like virus sequence was recombinant with any of the other related viruses, similarity plot analyses using the SIMPLOT package version 2.5 (Ray, 1999; http://www.med.jhu.edu/deptmed/sray) was performed on the nucleotide alignment with the new *BSRV-li*ke virus sequence and known *BSRV* and *BSRV-like* virus strains available in the NCBI GenBank with a sliding window of 200 nucleotides (nt) moving in steps of 50 nt. We excluded a total of 190 nt from divergent positions, mainly due to nucleotide (nt) insertion and/or deletion linked to the *RhPV* strains from this alignment, as well as the 5′ and 3′ UTR. Overall, the removed nucleotides represented 2.1% of the ORF1-IGR IRES-ORF2 genome with a total 52 nt removed from the ORF-1—55 from the IGR-IRES and 87 from the ORF-2, respectively. For the latter, 84 nt were removed only from the *RhPV* strains at the 3′ part of the ORF-2 due to the longer *RhPV* sequences than the other viruses. Actually, the adjustment realized for this alignment did not impact the SIMPLOT analysis.

### 2.11. GenBank Accession Numbers

The metagenomic data have been deposited to the Sequence Read Archive (SRA) under the accession number PRJNA574024.

The GenBank accession numbers of BSRV-like CR-026, ALPV CR-026, and ABPV CR-026 full genome sequences are MN510867, MN510868, and MN510869, respectively.

## 3. Results

### 3.1. Diet Composition

Faeces samples (CR-026; tubes 16 and 17)) were also analyzed. The sequencing and initial sequence validation yielded to a mean of 9627 and 8257 reads. These reads produced 294 and 216 distinct sequences. Sequences presenting less than five reads were discarded, and the remaining sequences were clustered into 41 and 29 MOTUs that were compared to BOLD [43]. According to the sample, the final, complete dataset represented a panel of 14 and 11 identified arthropods. They mainly correspond to lepidopters, which are consumed by the bat we studied. However, two dipters of the genera *Protoclythia* and *Hydrotaea* were also identified. These specimens could correspond to *Protoclythia modesta* and *Hydrotaea scambus,* but these identifications at the species level have to be considered with caution, as the percentage of matching with the sequences available in the public databases is not maximal (97.5% and 98.7%, respectively). This result is probably linked to the important lack of African arthropod sequences presently available in these databases.

### 3.2. Sequencing Results

The raw sequence number obtained by the HTS was 8,680,414 reads. Indeed, 161,815 reads belonged to the *Dicistroviridae* viral family, representing 30.8% of the viral reads obtained. The majority of the *Dicistrovirus* sequences generated (161,019 reads) were close to the *Cripavirus* genus, whereas 796 reads were close to the *Aparavirus* genus. By using De Novo Assemble command in Geneious Prime software, we obtained 13 contigs with a nucleotide range from 156 to 9858 bases. By using NCBI-BLAST (https://blast.ncbi.nlm.nih.gov/Blast.cgi) with default parameters (Megablast) and a “nucleotide” database, one contig (9983 nt) matched with the *Big Sioux River Virus* (*BSRV*) with a percentage of similarity of 91%, five contigs of size 5458, 2478, 1498, 334, and 247 nt matched with the *Aphid Lethal Paralysis Virus* (*ALPV*) with a percentage of similarity of 95.6%, 98%, 97%, 95.2%, and 97.6% for each contig, respectively, and seven contigs of size 4289, 1903, 1745, 1082, 759, 574, and 536 nt matched with the *Acute Bee Paralysis Virus* (*ABPV*) with a percentage of similarity of 85%, 82.6%, 84.3%, 91%, 86%, 80%, and 86.4% for each contig, respectively. Then, the different contigs were mapped onto the adapted GenBank reference sequences (*ABPV*-AF486073; *ALBV*-MF458893; *BSRV*-KY933259) using Geneious Prime software.

### 3.3. Genome Recovery

The *BSRV*-like virus genome was fully sequenced with a depth of coverage of X 7433 and a full complete sequence length of 9983 nucleotides. *ABPV* was partially sequenced with a depth coverage of X 79. Three gaps of 290 bp, 445 bp, and 310 bp, respectively, were recovered by using specific primers. The *ALPV* was also partially recovered with a depth of coverage of X 17 and a gap of 70 bp. For *ABPV* and *APLV*, the gaps were filled using Reverse transcription with random hexamer primers, followed by a nested PCR using specific primers. The presence of the *BSRV-like virus* was also confirmed by Reverse transcription with random hexamer primers, followed by a nested PCR using specific primers on the RNA extracted from the faecal pool sample. The specific primers used are shown in the Supplementary Materials, Text S4. All amplicons were then purified and sequenced using the Sanger method. Finally, we generated three full genomes with a length of 9983 nt for *BSRV*, 9744 nt for *ALPV,* and 9525 nt for *ABPV,* respectively.

The three viruses—the *BSRV-like virus*, *ALPV,* and *ABPV*—showed all the genetic features previously described for the *Dicistroviridae* family, including two ORF separated by an IGE-IRES for which the nucleic acid’s length was consistent with that described for such viruses (*BSRV-like virus* = 597 nt, *ALPV* = 199 nt, and *ABPV* = 184 nt).

### 3.4. Phylogenetic Analyses

**Figure 1:**
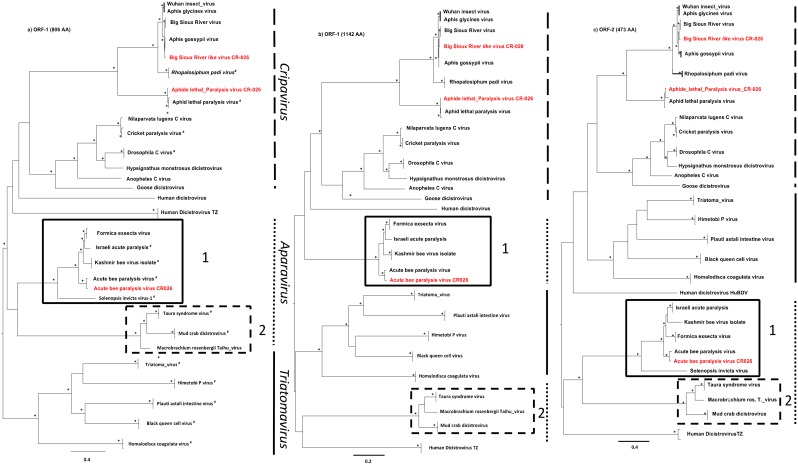
Phylogenetic relationships between *BSRV-li*ke CR-026, *ALPV* CR-026, and *ABPV* CR-026 with other representative *Dicistroviruses*. Phylogenies were inferred using Maximum Likelihood methods implemented in PhyML under the LG+Γ_4_+I model of evolution. Stars at nodes represent bootstrap (≥70%) values. Scale bars indicate substitution per site. The new *Dicistrovirus* strains are highlighted in red. Figure 1**a** represents the phylogenetic topology of ORF-1, including *Solenopsis invicta virus-1*; Figure 1**b** represents the phylogenetic topology of ORF-1, excluding the *Solenopsis invicta virus-1*; Figure 1**c** represents the phylogenetic topology of ORF-2. Viruses in the bold rectangle belong to the Aparavirus-1 genus, whereas viruses in the dashed rectangle belong to the aparavirus-2 genus. The vertical lines represent the different *Dicistrovirus* genera. The # symbol represents *Dicistroviruses* currently recognized by the ICTV. The *Dicistrovirus* reference strains used for these analyses are listed in the Supplementary Materials, Text S5.

To compare the three new *Dicistroviruses* obtained in this study to previously characterized *Dicistroviruses*, we performed phylogenetic tree analyses on both ORF-1 and ORF-2 amino acid sequences. For the ORF-1 we generated two different alignments. The first one included the *Solenopsis invicta virus*-1 (*SINV*-1), which belongs to the *Aparavirus* genus, and the second without this virus. The first 5′ part of *SINV-1* is considerably shorter than the other dicistroviruses. Thus, the non-use of *SINV-1* in the alignment allowed us to recover more than 300 AA representing >30% of supplementary genetic information within the ORF-1 (Figure 1a,b). Indeed, the tree topologies differed between these two alignments. The phylogenetic tree, including *SINV-1,* presented a cluster with two clades defined as *Aparavirus-*1 and *Aparavirus-2* [44], whereas without *SINV-1,* this cluster split into two different clusters, suggesting a more complex evolutionary history within the *Aparavirus* genus (Figure 1a,b) than previously described. Actually, the *Aparavirus* clade in the ORF-1, including *SINV-1,* is not well-sustained with a bootstrap value of solely 340/1000 which expresses a non-resolved phylogeny within the *Aparavirus* genus, whereas in the ORF-2 the *Aparavirus* clade is well-sustained.

**Figure 2:**
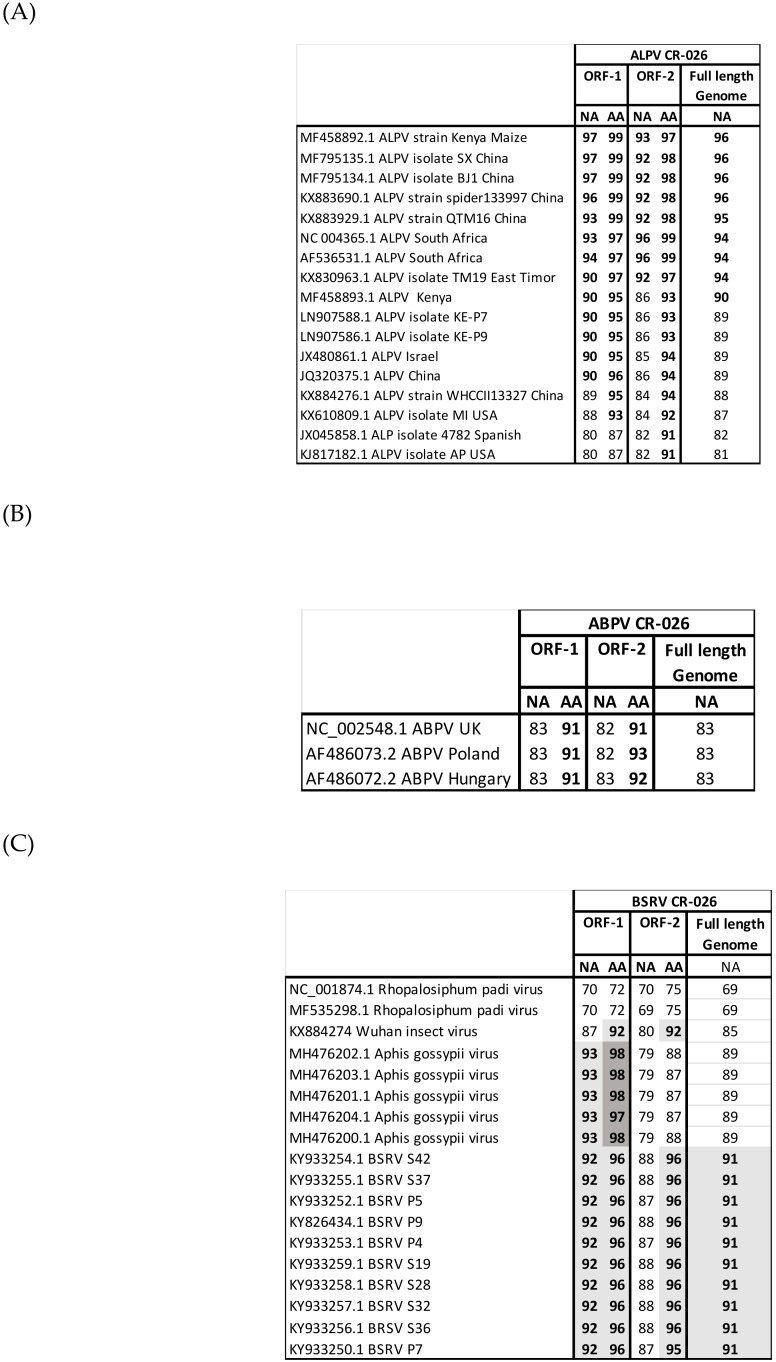
**Nucleotide and amino acid identities between**: (**A**) Nucleotide acid and amino acid identities between *ALPV CR-026* and other *ALPV* full-length genomes previously reported; (**B**) Nucleotide identities and amino acid between ABPV CR-026 and other *ABPV* full-length genomes previously reported; and (**C**) Nucleotide and amino acid identities between BSRV CR-026 and viruses belonging in the same phylogenetic cluster. Grey boxes highlight the NA and AA identities between BSRV CR-026 and closer viruses.

The *APLV* CR-026 and *ABPV* CR-026 identified in this study clustered with the previously characterized *ABPV* and *ALPV* (Figure 1). Full-length genome genetic distance analyses showed that *ALPV* CR-026 shared 96% of nucleotide identities with the *ALPV* KE P9 strain recently identified from maize leaves in Kenya [12] and with different *ALPV* strains characterized in China (Figure 2A). Only three *ABPV* complete genomes were available in the GenBank. *ABPV* CR-026 presented 83% of nucleotide identity and 91 to 93% of amino acid identities with these viruses (Figure 2B), which together shared 94 to 97% of nucleotide identities.

*BSRV-Like CR-026* clustered with the previously described *BSRV* [12], as well as with the *Wuhan insect virus* (*WIV*) [22] and *Aphis glycines virus* (*AGLV*) [18] characterized in China and *Aphid gossypii virus* (*AGV*) isolated in Israel (Figure 1). Sequences of *AGLV* were available in the GenBank but with no publication associated. Altogether, these viruses grouped with the *Rophalosiphum padi virus* (*RhPV*) and *ALPV,* and formed the *Cripavirus* genus. Amino acid and nucleotide identities showed that *BSRV-Like CR-026* was closer to *AGV* in the ORF-1 and to *BSRV* in the ORF-2 (Figure 2C). Nonetheless, the difference between *BSRV like CR-026*, *BSRV,* and *AGV* in the 0RF-1 is very weak, suggesting a common origin of these three viruses.

**Figure 3:**
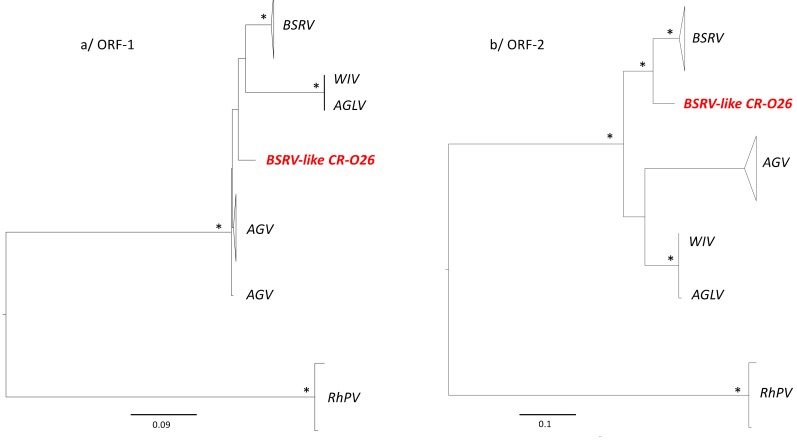
Phylogenetic relationships between *BSRV-like CR-026* with other representative *Cripaviruses* close to *BSRV* strains. Phylogenies were inferred using Maximum Likelihood methods implemented in PhyML under the LG+Γ_4_+I model of evolution. Stars at nodes represent bootstrap (≥70%) values. Scale bars indicate substitution per site. The new *BSRV-like CR-O26* strains are highlighted in red. Figure 2**a** represents the phylogenetic topology of ORF-1; Figure 2**b** represents the phylogenetic topology of ORF-2. *RhPV* was used as the out-group.

In order to further understand the relationship within this group of viruses, we proceeded by using ORF-1 and 2 amino acid sequences, as well as new phylogenetic analyses, including *BSRV*, *BSRV-like CR-026*, *AGV*, *WIV*, *AGLV,* and *RhPV* as the out-group. In the ORF-1, although the clade of *BSRV*, *BSRV-like CR-026, AGV, WIV*, and *AGLV* was phylogenetically well-sustained, the positioning of the different viruses in this clade remained unclear and the relationship between this group of viruses has yet to be disentangled (Figure 3a). In the ORF2, *BSRV-like CR-02*6 clustered with the previously reported *BSRV,* and could represent a new *BSRV* sub-type (Figure 3b). 

**Figure 4:**
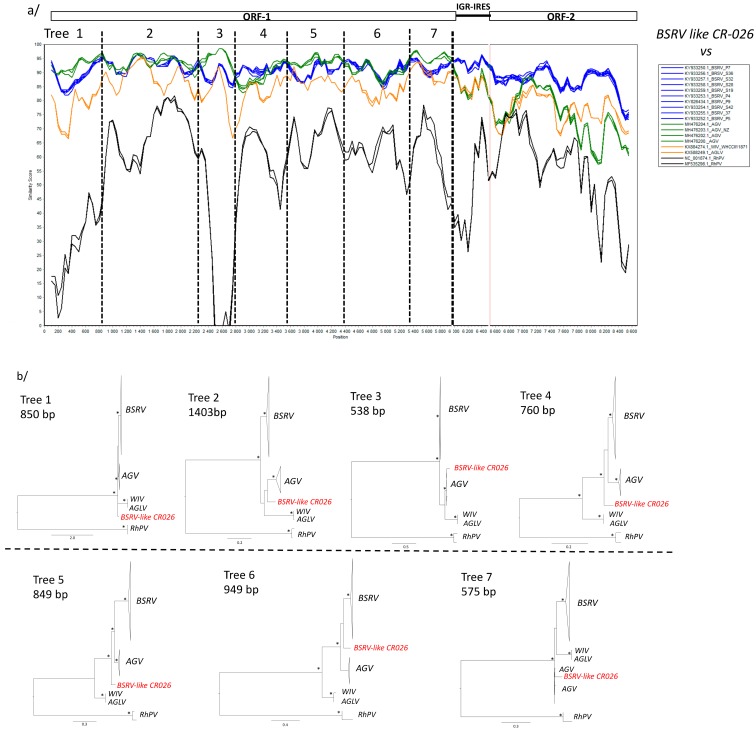
SIMPLOT analyses of the new *BSRV-like CR-026* whole genome sequences versus other *Cripavirus* lineages. (**a**) SIMPLOT analyses were performed using the full-length nucleic acid alignment of the *Cripavirus* of interest with the SIMPLOT package version 2.5 with a sliding window of 200 nucleotides (nt) moving in steps of 50 nt. Blue lines represent *BSRV* strains, orange lines represent *WIV* strains, green lines represent AGV strains, and black lines represent *RhPV* strains. Vertical black dash lines represent the partitions used for further phylogenetic analyses. (**b**) Phylogenetic analysis of partitioned ORF-1 nucleotide acid sequence based on the SIMPLOT analysis. Phylogenies were inferred using Maximum Likelihood methods implemented in PhyML under the LG+Γ_4_+I model of evolution. Stars at nodes represent bootstrap (≥70%) values. Scale bars indicate substitution per site. The new *BSRV-like CR-O26* strains are highlighted in red. *RhPV* was used as the out-group.

The SIMPLOT analysis confirmed our phylogenetic observations (Figure 4a). However, in the SIMPLOT analysis we observed that in the ORF-1, the new *BSRV-like CR-026* strain was closer to the *AGV* strains than other viruses in some parts of the nt sequence. In order to disentangle these complex phylogenetic relationships, we provided sequential phylogenetic analyses of the ORF-1 based on the SIMPLOT analysis (Figure 4b). This sequential analysis showed that *BSRV-like CR026* was positioned at the root of the *BSRV* and *AGV* clade in the trees 1, 4, 5. In the trees 2, 3, and 7, *BSRV-like CR026* were clustered with *AGV,* but the clades were not well-sustained, except for tree 3. In tree 4, the position of the *BSRV-like CR026* was also unresolved (Figure 4b).

**Figure 5:**
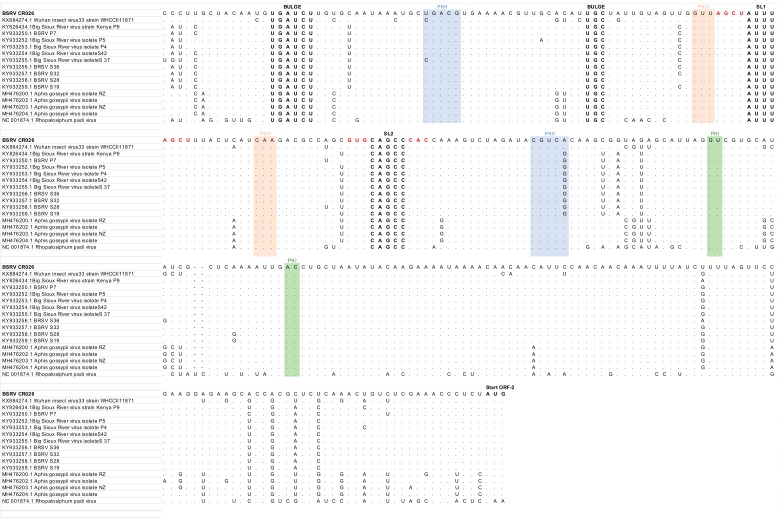
Comparison of class I IGRs of the *Cripavirus* genus.

Finally, we compared the IRES sequences of *BSRV*, *BSRV-like CR-026*, *AGV*, *WIV*, *AGLV,* and *RhPV* in order to confirm their close relationship. Data indicates that they all presented a similar genetic structure with two identical bulges and stem loops (SL1 and SL2), as well as similar pseudo-knots (PK1, 2, 3) (Figure 5) [1].

The IGRs were grouped into two classes, I and II. The *Cripavirus*’ IGRs belonged to the class I. The predicted base pairing was adapted from Jan et al. [1]. Bulges and stem loops are in bold. Stem loops predicted base pairing, and inverted repeat complementary sequences have been color-coded in red. Base pairing that formed pseudoknots PKI, PKII, and PKIII have been color-coded in green, blue, and orange, respectively. Conserved nucleotides have been replaced by dots, except for bulges and stem loops where letters are shown.

## 4. Discussion

Although viruses belonging in the *Dicistroviridae* family have been described worldwide, only a few studies have been carried out in African mainland [12,23,29,30,45,46,47]. In this study, we described the full-length sequences of two *Cripavirus* (*BSRV-like* and *ALPV*) and one *Aparavirus* (*ABPV*) obtained from the faeces of insectivorous bat species (*Hipposideros caffer*) collected in a cave dwelling in Zimbabwe.

Up to now, in African mainland, the *ALPV* full genome has been characterized only in South Africa and Kenya [12,48], whereas the full genome of the *BSRV-like* virus was first described in Kenya [12]. As for other *dicistroviru*s, very few data exist on *ABPV* in Africa, and only three full genomes are available in GenBank—one from South Africa [49], and two from Poland and Hungary [50].

*Dicistrovirus* can cause dramatic damage to the agriculture sector. For example, numerous *Dicistrovirus*, likely combined with other factors, have been associated with honeybee colony losses (Collapse Disorder Colony (CDD)) [8]. The *ABPV* is one such virus associated with this syndrome. In Zimbabwe, beekeeping is a growing farming activity. According to the Beekeepers Association of Zimbabwe, the country reached nearly 50,000 beekeepers who produced more than 1000 tons of honey per year (informal information). This activity represents a life-sustaining source of income and livelihood for small-scale farmers [51]. The characterization of *ABPV* in Zimbabwe’s insectivorous bats suggested a broader circulation of this virus in the country and probably in the rest of southern Africa. Additional studies need to be carried out in Zimbabwe to assess the prevalence of *ABPV* in beehives and to identify concomitant factors likely involved in CCD, such as Varroa’s mite infections [8,52], considering that the presence of *Dicistroviru*s could be a major concern for the agricultural sector in future.

On one hand, some *Dicistroviru*s are studied for their use as potential bio-pesticides [3]. Aphids are key pests of many important crops worldwide [53], and the *ALPV* is one of the recognized dicistroviruses which are pathogenic to insect pests of agricultural importance [3,54]. In Zimbabwe, agriculture, particularly tobacco farming, represents the most valuable economic agricultural income for the country, and the sector provides employment for up to 70 percent of the population. Chemical aphicides are preferentially used to control crop aphid invasion. The over- or misuse of chemical aphicide can led to aphid resistance, causing important yield losses [55]. Actually, bio-pesticides using lethal aphid viruses or natural predators could offer interesting alternatives to aphid proliferation control.

*BSRVs* have been described in diverse arthropod hosts worldwide [12,18,21,22]. Nonetheless, their pathogenicity in hosts is yet to be clarified. The *BSRV* taxonomic classification by the ICTV, as many other dicistroviruses, is still pending [2]. From our results, *BSRV-like CR-026* seemed to be a new *Cripavirus* based on the first part (ORF-1) of its genome, whereas the second part (ORF-2) of its genome suggests that it is close to the *BSRVs* previously characterized. Nonetheless, according to the ICTV, the species demarcation criteria within the *Cripavirus* is defined by the sequence identity at the amino-acid level between the capsid proteins of isolates and strains. Isolates with AA identities above 90% should be considered as the same species. Regarding this criterion, our new *BSRV* should be fully considered as a *BSRV* strain. However, considering the ORF-1 analyses, the taxonomic classification of *BSRV-like CR026* remains difficult to establish. Besides the *WIV* and *AGLV* showed 100% of AA identities in both ORF-1 and ORF-2 (Capsid proteins) and have 91% of AA identities with the BSRV strains. Obviously, these two viruses, *WIV* and *AGLV*, are identical and belong to the BSRV strains (see Supplementary Materials, Table S6). 

Recently, the *Dicistrovirus* has been reported in blood samples of human and bat species [29,30,31]. Although the identification of such viruses in humans was not clearly linked with their febrile state, these findings highlighted the potential spillover of such viruses in mammals and the need to identify its origin, particularly in the context of close contact between humans and bats, as observed in the current study site, and in all countries where bat guano is collected as fertilizer.

Finally, this study showed the potential of insectivorous bat faeces for the study of arthropod viromes. Insectivorous bats are generalized predators and they feed on a large number of insect species, following their availability as prey within the range of the insectivorous bat species [56,57]. As a colony of 300 insectivorous bats can consume around 2 to 6 million insects per year [57], insectivorous bats can be considered as a good collector of arthropod samples. Nonetheless, the *Hipposideros caffer* bat species is considered a moth specialist. Its diet consists predominantly of Lepidoptera [34,58,59]. In this study, we also checked the prey eaten by the *H. caffer* in our study site. Our result confirmed the previous reports with a majority of Lepidoptera found in the faeces, but also of dipters (see Supplementary Materials, Figure S2). The absence of Hymenoptera in our analysis suggested that the *Dicistrovirus,* such as the *Acute Bee Paralysis Virus,* can be hosted by other arthropods, and highlighted the need to further document the potential insect reservoirs of such viruses.

## Supplementary Materials

The following are available online at https://www.mdpi.com/1999-4915/11/12/1102/s1, Figure S1: Phylogenetic analysis of partial bat *CytB* sequences (550 bp), Figure S2: Order and family of insect found in the faeces’ bats by NGS, Text S3: Command lines used for bioinformatic pre-analysis, Text S4: Specific primers used to fill the gap and to confirm the presence of Dicistrovirus in the feces samples, Text S5: Dicistrovirus reference strains used for the phylogenetic analyses presented in Figure 1, Table S6:Amino acid identities between *BSRV*, *AGV*, *WIV* and *AGLV*
