# Supplementary material for: Next-Generation Sequencing on Insectivorous Bat Guano: An Accurate Tool to Identify Arthropod Viruses of Potential Agricultural Concern
Source: Viruses. 2019 Nov 28;11(12):1102. doi: 10.3390/v11121102 (PMC6950063; doi:10.3390/v11121102)
Supplement: Supplementary file 1 [file viruses-11-01102-s001.pdf]

Supplementary Materials

Figure 1S:

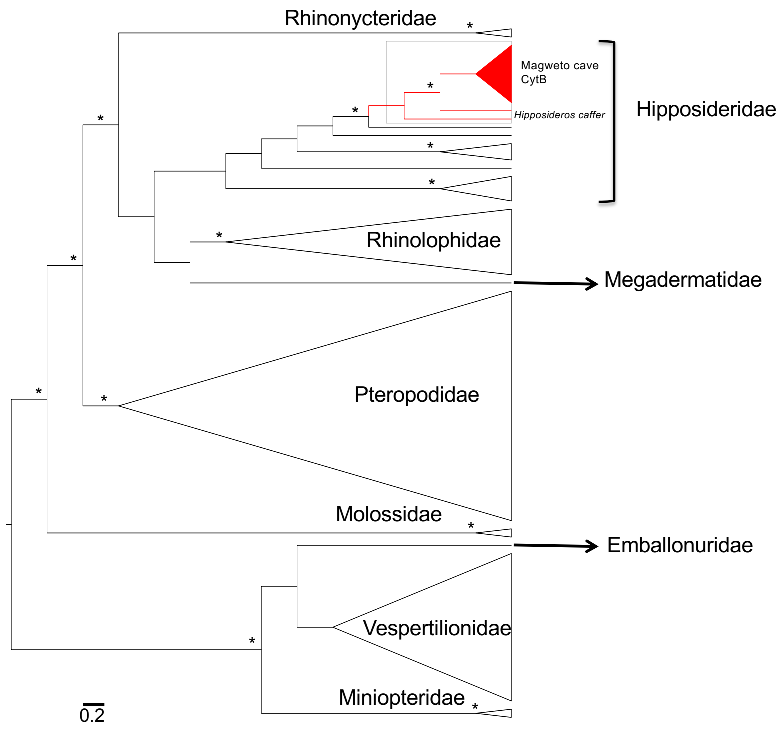

Phylogenetic analysis of partial *CytB* sequences (550 bp). New *CytB* sequences obtained were compared to representative bat family *CytB* sequences available in the GenBank. Only Bayesian posterior probabilities are showed. Asterisks at nodes represent posterior probability ≥85%. Scale bars indicate the number of base substitutions per site. New *Hipposideros caffer* CytB sequences are represented by the red triangle. The following sequences have been used for this analysis: KX467590.1 *Miniopterus schreibersii*, KX467591.1 *Miniopterus magnater*, KX467593.1 *Tylonycteris pachypus*, KX467595.1 *Hypsugoal aschanicus*, KX467596.1 *Nyctalus plancyi*, KX467597.1 *Pipistrellus abramus*, KX467598.1 *Murina leucogaster*, KX467601.1 *Myotis laniger*, KX467611.1 *Myotis davidii*, KX467606.1 *Myotis blythii*, KX467607.1 *Myotis chinensis*, KX467608.1 *Myotis pequinius*, KX467609.1 *Myotis bombinus*, KX467605.1 *Myotis formosus*, KX467610.1 *Myotis altarium*, KX467612.1 *Myotis frater*, KX467599.1 *Myotis ricketti*, KX467600.1 *Myotis adversus*, KX467604.1 *Myotis fimbriatus*, KX467602.1 *Myotis daubentonii,* KX467603.1 *Myotis macrodactylus*, KF218429.1 *Taphozous nudiventris*, AY591536.1 *Otomops martiensseni*, AY591537.*1 Otomops martiensseni*, KX467574.1 *Megaderma lyra*, KX467575.1 *Rhinolophus pusillus*, KX467576.1 *Rhinolophus lepidus*, KX467579.1 *Rhinolophus macrotis*, KX467580.1 *Rhinolophus marshalli*, KX467582.1 *Rhinolophus rex*, KX467578.1 *Rhinolophus ferrumequinum*, KX467583.1 *Rhinolophus sinicus*, KX467577.1 *Rhinolophus affinis*, KX467581.1 *Rhinolophus pearsonii*, KX467584.1 *Hipposideros cineraceus*, KX467585.1 *Hipposideros pomona*, FJ347979.1 *Hipposideros caffer*, FJ347980.1 *Hipposideros caffer*, KX467587.1 *Hipposideros armiger*, KX467588.1 *Hipposideros larvatus*, KX467589.1 *Hipposideros pratti*, KX467586.1 *Aselliscus stoliczkanus*, KT583805.1 *Hipposideros vittatus*, KT583806.1 *Hipposideros vittatus*, KT583801.1

Hipposideros gigas, KT583817.1 *Hipposideros commersoni*, DQ005796.1 *Triaenops persicus*, DQ005797.1 *Triaenops persicus*, KX823312.1 *Megaerops niphanae*, KX823313.1 *Cynopterus sp.,* KX823314.1 *Cynopterus sp.*, KX823317.1 *Sphaeria sblanfordi*, KX822888.1 *Eidolon helvum*, KX822889.1 *Eidolon helvum*, KX823318.1 *Macroglossus sobrinus*, KX823319.1 *Eonycteris spelaea*, KX822938.1 *Rousettus aegyptiacus*, KX822939.1 *Rousettus aegyptiacus*, KX823315.1 *Rousettus leschenaultii*, KX823311.1 *Plerotes anchietae*, KX823011.1 *Hypsignathus monstrosus*, KX823012.1 *Hypsignathus monstrosus*, KX823087.1 *Epomops franqueti*, KX823088.1 *Epomops franqueti*, KX823233.1 *Epomops buettikoferi*, KX823234.1 *Epomops buettikoferi*, KX822985.1 *Nanonycteris veldkampii,* KX822986.1 *Nanonycteris veldkampii*, KX823306.1 *Epomops dobsonii*, KX823307.1 *Epomophorus anselli*, KX823308.1 *Epomophorus crypturus*, KX823309.1 *Epomophorus labiatus*, KX823310.1 *Epomophorus minimus*, KX822798.1 *Epomophorus gambianus*, KX822810.1 *Micropteropus pusillus*, KX822797.1 *Epomophorus gambianus*, KX822809.1 *Micropteropus pusillus*.

Figure2S: Order and family of insect found in the faeces' bats. The confident value for the taxonomic identification must be equal or superior to 0.98. In green are represented values between 0.98 and 1.

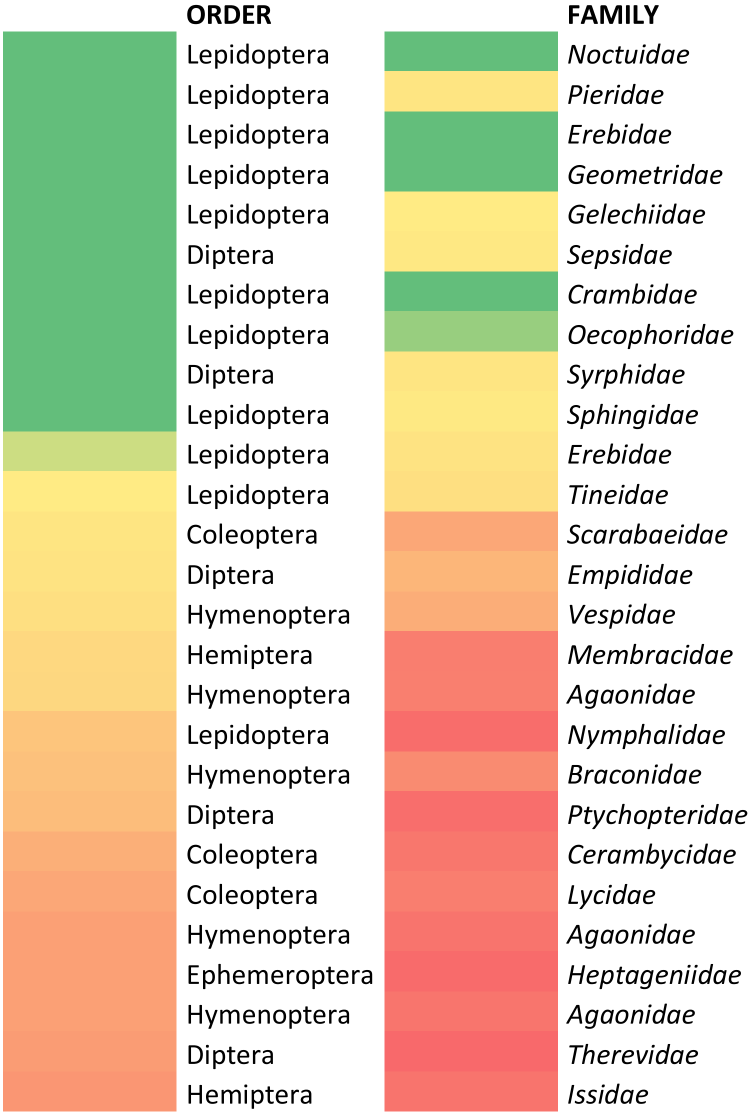

Text S3: Command lines used for bioinformatic pre-analysis

Database formatting

makeblastdb -in database/C-RVDBv12.2.fasta -dbtype nucl -out DB_RVDB

Querying database

blastn -query 1_S1_L001_R1_001.fasta -outfmt "6 qseqid sseqid qseq qlen pident nident mismatch evalue " -evalue 1e-9 -out reads1_RBDB.out -db DB_RBDB -num_threads 6

Reads indexing

makeblastdb -in 1_S1_L001_R1_001.fasta -parse_seqids -dbtype nucl -out DB_reads1

Reads ID list creation

```
grep -i "virus_name" reads1_RBDB.out | cut -f 1 | sort -u > reads_ID

### Reads extraction

blastdbcmd -entry_batch reads_ID -db DB_reads1 -dbtype nucl -out
virus_name_reads.fasta
```

Text S4: Specific primers used to fill the gap and to confirm the presence of Dicistrovirus in the feces samples.

Big Sioux River Virus specific primers

Amplicon size: 1531 bp
BSRV-F1-30 3'- GAGTGATTAACCCGCCTTTAT-5'
BSRV-R1-1523 3'-TTCCTGTAGCCTCCGATA-5'
Amplicon size: 616 bp
BSRV-F2-529 3'- TTTAGCTGCCTTAAGCAAC-5'
BSRV-R2-1127 3'- AAGCTCGGACGAGTCCAA-5'

Acute Bee Paralysis Virus specific primers
Fragment A
Amplicon size: 623 bp
A-F1 3'-CAGGATTTCGACAAACTTACTCA-5'
A-R1 3'-ACTCTTACTATCTAGTTCTCCCTC-5'
Amplicon size: 324 bp
A-F2 3'-GCAAAACCAACTATGAAAATTGCT-5'
A-R2 3'-TCCAGATTTACCTCGTCCTGATTCTCC-5'

Fragment B
Amplicon size: 1014 bp
B-F1 3'-TTGGGATAACTATCAAGGCCAGAAC-5'
B-R1 3'-TCACAAAGCCATTTCCAGAAGCCA-5'
Amplicon size: 748 bp
B-F2 3'-GATTCATCTTCAAATCCTAACCCTG-5'
B-R2 3'-GTTCCCATTGTTCTTCATAATC-5'

Fragment C
Amplicon size: 644 bp
C-F1 3'-TCACGTGCAGGGGTCACAGGATA-5'
C-R1 3'-CATTTGATCGTAGATGTTACGG-5'
Amplicon size: 484 bp
C-F2 3'-GTAGAAATGACGATACCATATGC-5'
C-R2 3'-TAGGTCCAATCTTAGGATTCTCA-5'

Aphid Lethal Paralysis Virus specific primers
Amplicon size: 347 bp
ALPV F1 3'-TAAGAATCCTGAACTGAAACGAC-5'
ALPV R1 3'-CGATCCTTTATCTTGTTCAGGT-5'
Amplicon size: 217 bp
ALPV F2 3'-TCTCGCCGTGCGAATTCAACT-5'
ALPV R2 3'-AGCAGGTACTGTTTGTATGTC-5'

Text S5: Dicistrovirus reference strains used for the phylogenetic analyses presented in Figure 1.

KX884274 *Wuhan insect virus*
MH476202.1 *Aphis gossypii virus*
MH476203.1 *Aphis gossypii virus*
KY826434.1 *Big Sioux River virus*
KY933256.1 *Big Sioux River virus*
NC001874.1 *Rhopalosiphum padi virus*
MF535298.1 *Rhopalosiphum padi*
MF458893.1 *Aphid lethal paralysis virus*
MF458892.1 *Aphid lethal paralysis virus*
KM270560.1 *Nilaparvata lugens C virus*

NC003924.1	*Cricket paralysis virus*
KP974706.1	*Cricket paralysis virus*
AF014388.1	*Drosophila C virus*
NC001834.1	*Drosophila C virus*
MH310078.1	*Hypsignathus monstrosus dicistrovirus*
KU169878.1	*Anopheles C virus*
NC029052.1	*Goose dicistrovirus isolate*
KF500001.1	*Formica exsecta virus*
NC023021.1	*Formica exsecta virus*
KY465696.1	I*sraeli acute paralysis virus*
KF500001.1	I*sraeli acute paralysis virus*
KF956377.1	*Kashmir bee virus*
AF486073.2	*Acute bee paralysis virus isolate*
AF178440.1	*Triatoma virus*
NC003783.1	*Triatoma virus*
NC003779.1	*Plauti astali intestine virus*
AB006531.1	*Plauti astali intestine virus*
NC003782.1	*Himetobi P virus*
KM270559.1	*Himetobi P virus*
MH267694.1	*Black queen cell virus isolate*
MH267693.1	*Black queen cell virus isolate*
NC008029.1	*Homalodisca coagulata virus*
KT207917.1	*Homalodisca coagulata virus*
JX094350.1	*Taura syndrome virus*
NC003005.1	*Tauras yndrome virus*
NC018570.2	*Macrobrachium rosenbergii Taihu virus*
HQ113110.2	*Macrobrachium rosenbergii Taihu virus*
NC014793.1	*Mud crab dicistrovirus*
HM777507.1	*Mud crab dicistrovirus*
AY634314.1	*Solenopsis invicta virus*
KY973643.1	*Human Blood-associated Dicistrovirus*
MH536109	*Human Dicistrovirus*
MH536110	*Human Dicistrovirus*
KX588249.1	*Aphis glycines virus*

Table S6: Amino acid identities between *BSRV*, *AGV*, *WIV* and *AGLV*

ORF-1 and ORF-2 Amino acid identities between *BSRV*, *AGV*, *WIV* and *AGLV* are showed. Species demarcation criteria within the *Cripavirus* genus is amino acid identities of the capsid protein (ORF-2) above 90%.

	BSRV CR026	BSRV P7	BRSV S36	BSRV S32	BSRV S28	BSRV S19	BSRV P4	BSRV P9	BSRV S42	BSRV S37	BSRV P5	AGV	AGV NZ	AGV	AGV AB	AGV RZ	WIV	AGLV
BSRV CR026	-	96	96	96	96	96	96	96	96	96	96	98	98	98	98	98	92	92
BSRV P7	**96**	-	100	100	100	100	100	100	100	100	100	96	96	96	96	96	91	91
BRSV S36	**96**	**100**	-	100	100	100	100	100	100	100	100	96	96	96	96	96	91	91
BSRV S32	**96**	**100**	**100**	-	100	100	100	100	100	100	100	96	96	96	96	96	91	91
BSRV S28	**96**	**99**	**100**	**100**	-	100	100	100	100	100	100	96	96	96	96	96	91	91
BSRV S19	**96**	**100**	**100**	**100**	**100**	-	100	100	100	100	100	96	96	96	96	96	91	91
BSRV P4	**96**	**100**	**100**	**100**	**100**	**100**	-	100	100	100	100	96	96	96	96	96	91	91
BSRV P9	**96**	**99**	**100**	**100**	**100**	**100**	**100**	-	100	100	100	96	96	96	96	96	91	91
BSRV S42	**96**	**100**	**100**	**100**	**99**	**100**	**100**	**99**	-	100	100	96	96	96	96	96	91	91
BSRV S37	**96**	**100**	**100**	**100**	**100**	**100**	**100**	**100**	**100**	-	100	96	96	96	96	96	91	91
BSRV P5	**96**	**100**	**100**	**100**	**100**	**100**	**100**	**100**	**100**	**100**	-	96	96	96	96	96	91	91
AGV	87	87	87	87	87	87	87	87	87	87	87	-	100	100	100	99	92	92
AGV NZ	87	87	87	87	87	87	87	87	87	87	87	**98**	-	100	100	99	92	92
AGV	88	87	88	88	88	88	88	88	87	88	88	**98**	**100**	-	100	100	92	92
AGV AB	87	87	88	87	88	88	87	87	87	88	87	**99**	**100**	**100**	-	100	92	92
AGV RZ	87	87	88	87	88	88	87	87	87	88	87	**99**	**100**	**100**	100	-	92	92
WIV	**91**	**91**	**91**	**91**	**91**	**91**	**91**	**91**	**91**	**91**	**91**	89	89	90	89	89	-	**100**
AGLV	**91**	**91**	**91**	**91**	**91**	**91**	**91**	**91**	**91**	**91**	**91**	89	89	90	89	89	**100**	-

ORF-1

Capsid protein
